# Gut microbiota of preterm infants in the neonatal intensive care unit: a study from a tertiary care center in northern India

**DOI:** 10.3389/fmicb.2024.1329926

**Published:** 2024-02-08

**Authors:** Prabavathi Devarajalu, Jogender Kumar, Sourabh Dutta, Savita Verma Attri, Jayakanthan Kabeerdoss

**Affiliations:** ^1^Pediatric Biochemistry Unit, Department of Pediatrics, Post Graduate Institute of Medical Education & Research (PGIMER), Chandigarh, India; ^2^Newborn Unit, Department of Pediatrics, Post Graduate Institute of Medical Education & Research (PGIMER), Chandigarh, India

**Keywords:** preterm infants, probiotics, necrotizing enterocolitis, gestational age, India

## Abstract

**Introduction:**

Disruptions of the gut microbiota of preterm infants admitted to the neonatal intensive care unit (NICU) during the first 2 weeks of life are of critical importance. These infants are prone to various complications, including necrotizing enterocolitis (NEC) and sepsis. Studying the gut microbiota will improve outcomes in preterm infants. In the present study, we examined the gut microbiota of preterm infants admitted to the NICU in the first month of life.

**Methods:**

Neonates admitted to the NICU were recruited, and stool samples were collected weekly from the seventh day of the infant’s life until the 30th day of life. DNA was extracted using a DNeasy Powersoil DNA isolation kit. 16S rRNA gene sequencing targeting the V3–V4 region was performed using the MiSeq platform. Sequenced reads were processed on DADA2 pipeline to obtain an amplicon sequence variant (ASV) table. All bioinformatic and statistical analyses were performed using different packages in the R statistical framework.

**Results:**

Fourteen preterm infants were recruited, and 48 samples were collected. Alpha diversity metrics, observed ASV count, and Shannon index were found to have no differences in any clinical variables. Permutational multivariate analysis of variance (PERMANOVA) showed discrimination of neonates by gestational age and administration of probiotics. Differential abundance analysis showed a decreased abundance of *Bifidobacterium Breve* in extremely preterm infants (gestational age <28 weeks) compared to moderate preterm infants (gestational age 29–32 weeks). Supplementation with probiotics decreased *Acinetobacter* and increased *Bifidobacterium* in the gut of preterm neonates regardless of gestational age.

**Conclusion:**

Gestational age and probiotic supplementation alter the gut microbiota of preterm infants admitted to the NICU.

## 1 Introduction

Preterm infants have an immature gut and immune system. Several factors, including mode of delivery, sex, breast milk, intrapartum antibiotics, and antenatal corticosteroids, have been shown to alter preterm microbiota.

Delayed intestinal bacterial colonization and frequent antibiotic exposure in the early neonatal period cause the acquisition of antibiotic-resistant bacteria among preterm infants admitted to the neonatal intensive care unit (NICU) (Bargheet et al., 2023). Transmittance of pathogenic bacterial species occurs through apparatuses used in the NICU, environmental surfaces and nursing workspaces (Vongbhavit et al., 2022). Several studies showed that the NICU environment shapes the neonatal gut microbiota (Hartz et al., 2015; Brooks et al., 2018). All these factors contribute to the risk of sepsis and various degrees of systemic inflammation as well as cause gut microbiota dysbiosis in premature neonates. These factors also increase the risk for the development of necrotizing enterocolitis (NEC) and increase mortality.

Gut microbiota of infants admitted to NICU are more complex in resource-poor settings of low- and middle-income countries (LMICs) (Dhaded et al., 2022). India contributes to a high preterm birth rate and mortality among LMICs. Studying the gut microbiota will help to improve outcomes and develop newer intervention strategies in preterm infants born in LMICs.

The aims of this study are (1) to evaluate the gut microbiota of extremely preterm infants and moderate preterm infants in the first month of life in the NICU and (2) to evaluate the impact of gestational age (GA), probiotic supplementation, and morbidities, including sepsis and NEC, on the gut microbiota of preterm infants.

## 2 Materials and methods

### 2.1 Data and sample collection

We recruited preterm infants born at gestational age between 26 and 32 weeks with a birth weight of less than 1500 grams and a postnatal age of less than 72 h. This study was conducted between September 2022 and March 2023. Infants with congenital malformations, expected life expectancy of less than a week, and lack of parental consent were excluded from the study. Demographic details of the mothers were extracted from medical records, and neonatal information were collected prospectively until discharge. Infants received the probiotic Darolac (*Lactobacillus acidophilus*, *Lactobacillus rhamnosus*, *Bifidobacterium longum*, and *Saccharomyces boulardii*). One gram of probiotics mixed with expressed breast milk was given to infants for an average of 10 days once food tolerance was achieved. None of the mothers participating in the study had administered probiotics during the recruitment period.

Once infants received probiotics administration, the following collected samples were labeled as probiotic groups. Samples from infants who had not received probiotics before or at the time of collection were labeled as the non-probiotics group.

Mothers were informed about the study before enrolling their babies, and sterile containers were provided for stool sample collection. Fecal samples were collected weekly from the seventh day until the 30th day in the NICU and neonatal nursery. Stool samples were collected in sterile containers and immediately stored in the freezer compartment of the refrigerator. Samples were transported to the laboratory within 24 h and stored at −80°C until further processing. This study was conducted in accordance with the Declaration of Helsinki. The research protocol was approved by the Institutional Ethics Committee (IEC).

### 2.2 DNA extraction and sequencing

Genomic DNA was extracted from 150 milligrams of stool samples using DNeasy PowerSoil Pro Kit (Qiagen Inc., Germany). DNA concentration and purity were assessed on 1% agarose gels. The DNA concentration was quantified using a Qubit DNA HS assay (Invitrogen, Cat. no. Q32854). First, amplicon generation was performed for the 16S rRNA region covering 1500 bp, followed by a second amplicon generation targeting the V3-V4 region (460 bp) in a nested PCR approach (Kameoka et al., 2021). PCR products were run on 2% agarose gel electrophoresis for detection. All amplified PCR products were then cleaned (1 × ) using AMPure XP beads (Beckman Coulter, Cat. no. A63882). DNA library preparation was performed using the NEBNext Ultra DNA Library Prep Kit for Illumina (New England Biolabs, Cat. no E7370L). All prepared libraries were verified for fragment distribution by a capillary electrophoresis instrument (5300 Fragment Analyzer System, Agilent). Pooled libraries were loaded onto the MiSeq platform (Illumina Inc., USA) for 16S rRNA gene sequencing in a 2 × 250 bp paired-end format.

Barcodes and adapters were removed from raw sequences, and demultiplexed fastq files were generated. Trimmed sequences were imported to the DADA2 sequence processing pipeline to generate an amplicon sequence variant (ASV) table (Callahan et al., 2016). Taxonomy was assigned to the ASVs using a naïve Bayes classifier against a SILVA database version 138. After ASV filtering, we obtained 4,356,624 sequencing reads belonging to 6370 ASVs. Each sample was rarefied to 90763 reads to obtain an even sampling depth and used for all downstream analyses. As part of data pre-processing, samples that had ASV taxa counts below 20% were removed, and the remaining samples were used for downstream analysis. Raw sequences were deposited at the NCBI Sequence Read Archive under the Bioproject accession number PRJNA1048725.

### 2.3 Microbiota diversity, composition, and statistical analysis

Alpha diversity metrics, including observed ASV and Shannon index, were calculated using the Phyloseq package (McMurdie and Holmes, 2013). A one-way ANOVA test was used to compare diversity indices between samples collected weekly. The Wilcoxon rank-sum test was used to compare diversity indices between the preterm, probiotic, and NEC groups. A phylogenetic tree was constructed using the DECIPHER and Phangorn (v.2.4.0) packages in R (Schliep, 2011; Murali et al., 2018). Beta diversity metrics, Bray-Curtis, Jaccard, and weighted UniFrac distances were calculated from centered log-ratio (CLR)-transformed ASV counts using the vegan package in R. Permutational multivariate analysis of variance (PERMANOVA) was performed to test statistical significance by fitting distance data and metadata with 999 permutations. Gut microbiota intergroup variability was visualized on the ordination plot using non-metric multidimensional scaling (NMDS) and a weighted UniFrac method to calculate the distance between samples.

Differential abundances of microbiota between groups were performed by linear discriminant analysis (LDA) effect size (LEfSe) analysis, and the DESeq2 package in R (Segata et al., 2011; Love et al., 2014). An alpha-value of 0.05 was chosen as the significance cutoff and Benjamini-Hochberg adjusted FDR *p*-values were used for the DESeq2 method. For LEfSe analysis, data were normalized for the CPM method, and the run_lefse function was performed in the microbiomeMarker package with a default setting of LDA 2. Statistically significant associations between microbial traits and clinical covariates were identified using Microbiome Multivariable Association with Linear Models (MaAsLin2) (Mallick et al., 2021). Analysis was adjusted for postnatal age, and a *q*-value threshold of 0.25 was considered significant. Bioinformatics analysis and statistical analysis were performed in R software using various packages, including DADA2, Phyloseq, Vegan, MicrobiomeMarker, and ggplot2.

## 3 Results

The demographic characteristics of the study participants are shown in Table 1. Fourteen preterm infants were recruited, and 48 stool samples were collected (Supplementary Figure 1 for graphical representation). All mothers received antenatal steroids. Only one received antibiotics before delivery and ceftazidime, imipenem, and amikacin were administered for a week. Three infants developed NEC and they were at stage 2A, 2B, and 3B at the time of enrollment.

**TABLE 1:** Demographic details of the subjects.

Variables	*n* = 14
Gestational age (week), mean ± SD	29.3 ± 1.9
Sex (male/female)	6/8
Birth weight (g), mean ± SD	1044 ± 290
No. (%) of vaginal delivery	5 (35.7%)
No. (%) of early-GA subjects (≤28 weeks)	6 (42.9%)
No. (%) of mothers with PPROM	4 (28.6%)
No. (%) of mothers with preeclampsia	5 (35.7%)
No. (%) of mothers receiving antenatal corticosteroids	14 (100%)
APGAR score at 1 min median (IQR)	7 (6–8)
APGAR score at 5 min median (IQR)	8 (7–9)
No. (%) of neonates receiving antibiotic	14 (100%)
No. (%) of infants with NEC	3 (21.4%)
No. (%) of infants with Sepsis	3 (21.4%)

PPROM, preterm premature rupture of membranes.

### 3.1 Alpha- and beta-diversity of preterm infants

**FIGURE 1:**
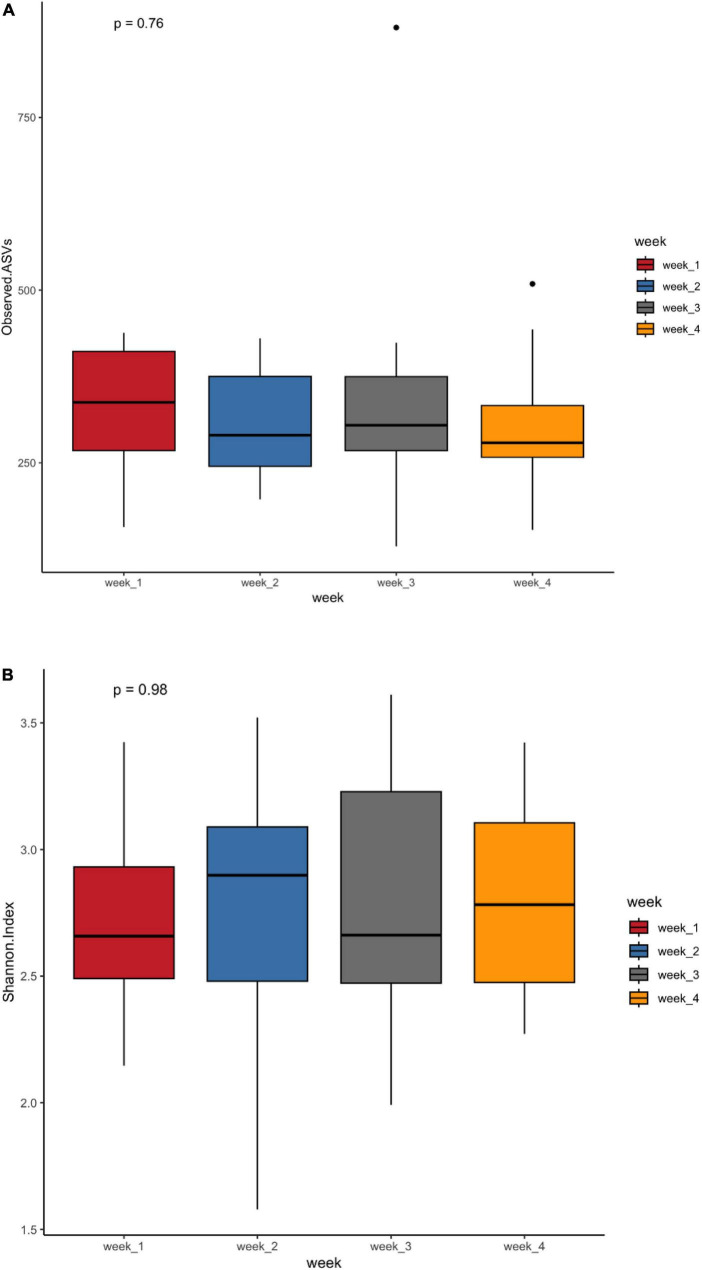
Alpha diversity metrics observed ASVs **(A)** and Shannon diversity **(B)** in the first 4 weeks of life of preterm infants.

The observed ASV and Shannon indices measured to assess microbial richness and evenness showed no difference between weeks (Figure 1). To assess the impact of microbiota changes on gestational age, preterm infants born between 26 and 28 weeks were classified as extremely preterm, and neonates born between 29 and 32 weeks were labeled as moderate preterm infants. The observed ASV and Shannon index values did not differ between extremely preterm and moderate preterm infants or between preterm infants with and without probiotic supplementation (Supplementary Figure 2). Although there was no statistical significance between the NEC and non-NEC groups, a trend could be observed in the Shannon index (Supplementary Figure 3). This indicates that preterm infants with NEC harbor more ASV and bacterial communities.

**FIGURE 2:**
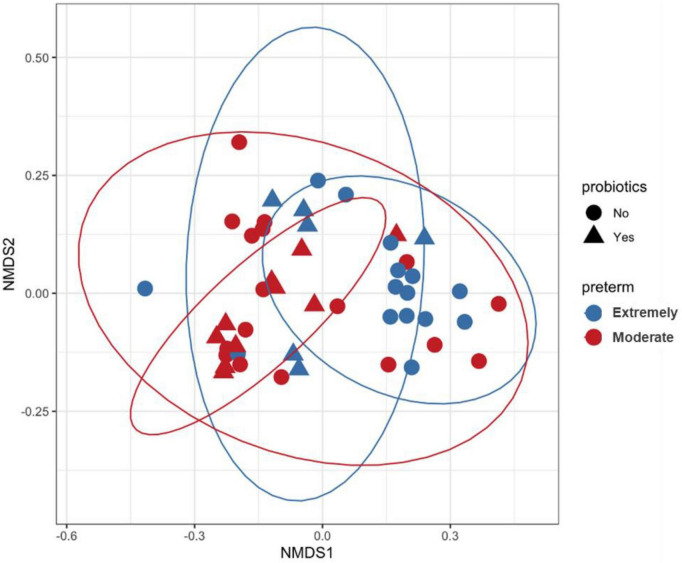
Non-metric multidimensional scaling (NMDS) plot generated using weighted UniFrac distances. Each sample is represented by one dot, blue color represents samples of Extremely pre-term infants, red color represents samples of moderate pre-term infants and shaped according to the administration of probiotics. Inner and outer circles separate the samples based on probiotic supplementation and gestational age, respectively.

PERMANOVA analysis on distances calculated from Bray-Curtis and Jaccard showed that microbiota composition in preterm infants was influenced by gestational age, probiotics and development of NEC (Table 2 and Supplementary Table 1). PERMANOVA based on the weighted UniFrac method showed significance for gestational age and probiotics but not for NEC (Supplementary Table 2). Non-metric multidimensional scaling (NMDS) plotted with weighted UniFrac distances probiotic supplementation discriminated samples based on gestational age and probiotic (Figure 2). Bray-Curtis and Jaccard were based on abundance (ASV count) and presence or absence of ASV, respectively. The weighted UniFrac method is based on phylogenetic diversity. Therefore, the results obtained from the Bray-Curtis and Jaccard measurements differ from the weighted UniFrac results for NEC.

**TABLE 2:** Permutational multivariate analysis of variance (PERMANOVA) analysis performed based on Bray-Curtis dissimilarity distance.

	Df	Sums of Sqs	MeanSqs	F. Model	*R* ^2^	Pr (>*F*)
Week	3	0.38978848	0.12992949	0.61482323	0.0315773	0.865
Probiotics	1	0.93290101	0.93290101	4.41446506	0.07557558	**0.006**
Preterm	1	0.87076903	0.87076903	4.12045804	0.07054219	**0.006**
NEC	1	0.4864281	0.4864281	2.30176605	0.0394062	**0.04**
Week: probiotics	3	0.61236171	0.20412057	0.96589362	0.04960826	0.467
Week: preterm	3	0.6767952	0.2255984	1.06752618	0.0548281	0.388
Probiotics: preterm	1	0.45692388	0.45692388	2.16215279	0.03701603	0.063
Week: NEC	2	0.57552906	0.28776453	1.36169481	0.04662439	0.191
Probiotics: NEC	1	0.54415463	0.54415463	2.57492657	0.04408271	**0.036**
Preterm: NEC	1	0.45902333	0.45902333	2.1720873	0.03718611	**0.025**
Week: probiotics: preterm	3	0.8354806	0.27849353	1.31782469	0.06768342	0.184
Week: probiotics: NEC	2	0.22058684	0.11029342	0.52190581	0.01787004	0.896
Residuals	25	5.2832053	0.21132821	NA	0.42799967	NA
Total	47	12.3439472	NA	NA	1	NA

Statistically significant variables are highlighted in bold.

### 3.2 Taxa associated with gestational age, probiotics, and NEC

**FIGURE 3:**
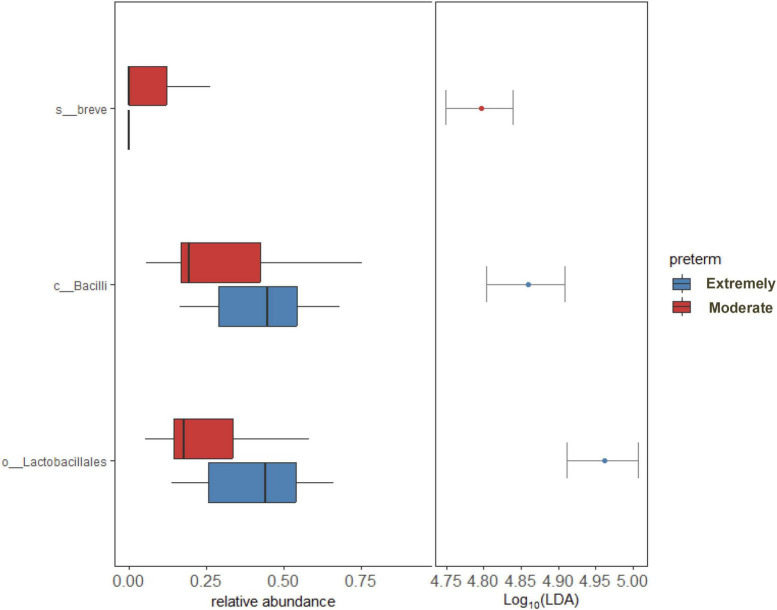
LEfSe analysis shows significant bacterial abundance between extremely preterm infants (26–28 weeks) and moderate preterm infants (29–32 weeks). Bacterial abundance shown only to significant corresponding Taxa level.

**FIGURE 4:**
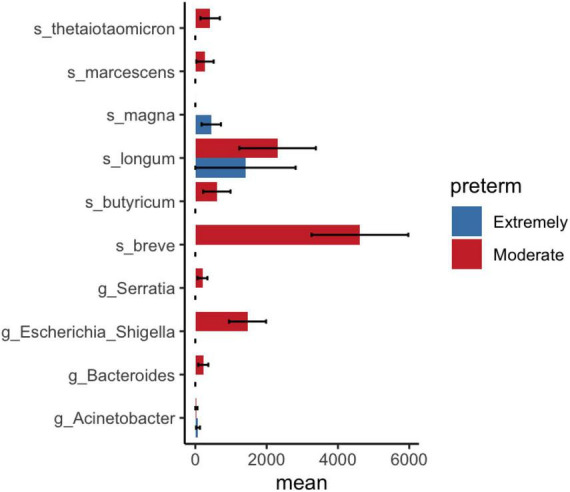
DESeq2 analysis shows significant bacterial abundance between extremely preterm infants (<29 weeks) and moderate preterm infants (29–32 weeks). Mean and standard error of ASV count shown in *x*-axis.

LEfSe and DESeq2 methods were used to identify microbial communities in significant variables in PERMANOVA. LEfSe analysis revealed a high abundance of *Bifidobacterium breve* and a low abundance of *Lactobacillales* at the order level in moderate preterm infants compared to extremely preterm infants (Figure 3). The DESeq2 method identified a high abundance of *Bifidobacterium breve*, *Bifidobacterium longum*, and *Bacteroides thetaiotaomicron* in the moderate preterm infant group, whereas an increased abundance of the *Finegoldia magna* and *Acinetobacter* genera was observed in extremely preterm infant neonates (Figure 4 and Supplementary Table 3).

**FIGURE 5:**
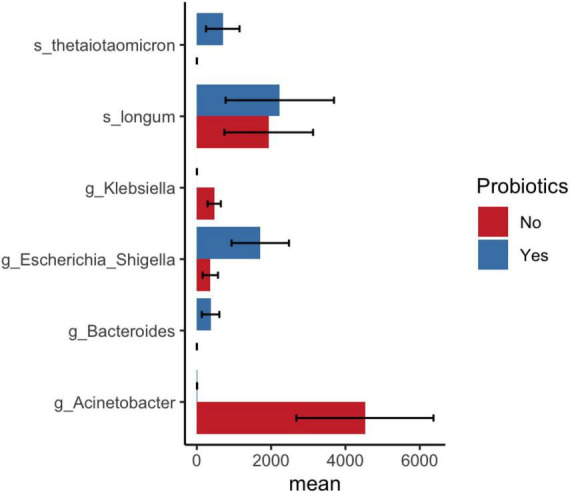
DESeq2 analysis shows significant bacterial abundance between samples after probiotic administration in infants and before/no probiotic administration in infant samples. Mean and standard error of ASV count shown in *x*-axis.

LEfSe analysis showed a significantly reduced abundance of the *Acinetobacter* genus and *Firmicutes* phylum and an increased abundance of *Enterobacterales* in probiotic-treated preterm infant samples compared with non-probiotic-treated samples (Supplementary Figure 4). Similar observations were noted in DESeq2 analysis, which revealed an increased abundance of *Bifidobacterium longum* and *Bacteroides thetaiotaomicron* as well as a decreased abundance of the genus *Klebsiella* in probiotic-supplemented samples compared to samples without non-probiotic samples (Figure 5 and Supplementary Table 4).

**FIGURE 6:**
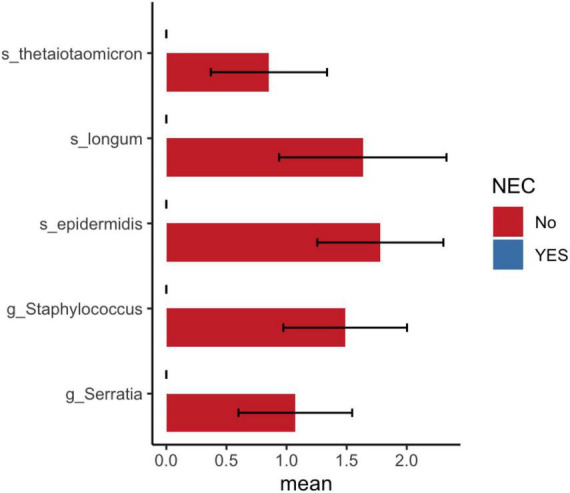
DESeq2 analysis shows significant bacterial abundance between preterm infants with and without NEC. Mean and standard error of log10 ASV count shown in *x*-axis.

Differential taxa analysis using LEfSe found significantly increased abundance levels of *Finegoldia magna* in NEC compared to non-NEC preterm infants (Supplementary Table 5). The DESeq2 results showed a decreased abundance of *Bifidobacterium longum*, *Bacteroides thetaiotaomicron*, *Staphylococcus epidermidis*, and *Serratia* genera in NEC compared to non-NEC preterm infants (Figure 6 and Supplementary Table 6).

A linear model multivariate analysis adjusted for postnatal age confirmed that both gestational age and probiotics significantly altered the gut microbiota of infants in our cohort (Supplementary Table 7). None of the covariates, including sepsis and NEC, were associated with bacterial counts. This confirmed the findings of LEfSe and DESeq2 analysis, which showed an decreased abundance of *Bifidobacterium breve* in extremely preterm infants compared to moderate preterm infants. Supplementation with probiotics increased *Lacticaseibacillus* and decreased the abundances of *Klebsiella* and *Acinetobacter* in preterm infants.

## 4 Discussion

Here, we evaluated factors affecting the gut microbiota of preterm infants admitted to the NICU in the first month of life. To the best of our knowledge, this is the first study to examine gut microbial communities in preterm infants in India. The present study found that the gestational age of preterm infants, supplementation of probiotics, and development of NEC modulated the gut microbiota of preterm neonates during the first month of life.

The results of the present study are consistent with previous observations that gestational age and probiotics are major determinants of gut microbiota maturation in preterm infants (Bargheet et al., 2023). Bacterial changes associated with gestational age could be due to the introduction of oral feeding, mainly mothers’ milk and donor milk. Human milk contains human milk oligosaccharides (HMOs), which are complex sugars that are substrates for the growth of *Bifidobacterium* and *Bacteroides* genera in the infant’s gut. Bifidobacterium increases the production of acetate and lactate, thereby decreasing luminal pH, which is one of the mechanisms to prevent fatal infections.

In our center, infants are fed probiotics mixed with expressed breast milk or donor milk. Both probiotics and human milk together increased the levels of *Bifidobacterium*. We did not find any association between nil per os (NPO) and the duration of NICU stay on microbiota changes using linear model multivariate analysis (data not shown). Thus, MaAsLin2 analysis showed that gestational age and probiotics independently altered the gut bacterial composition in preterm infants.

In India, *Acinetobacter baumannii* and *Klebsiella pneumoniae* are the most common pathogens detected in the organs of deceased premature infants (Ghanchi et al., 2023). These bacteria are associated with frequent nosocomial outbreaks in the NICU and are responsible for neonatal morbidity and mortality. In our study, administration of probiotics showed a reduction in *Acinetobacter* and *Klebsiella* counts. We have shown that probiotic supplementation increases the abundance of *Bifidobacterium longum* and *Bacteroides thetaiotaomicron*. These bacterial species were decreased in infants with NEC compared to those without NEC. Both bacteria have genes for the catabolism of HMO and are mutualistic in the infant gut (Marcobal et al., 2011). Monocolonization of *B. thetaiotaomicron* promotes intestinal microvasculature through Paneth cells (Stappenbeck et al., 2002). Microbial communities in the preterm gut modulate intestinal maturation and differentiation of epithelial cell lineages (Yu et al., 2016). Thus, probiotic supplementation promotes the enrichment of beneficial microbes in preterm neonates admitted to the NICU.

Commensal gut bacteria, *Finegoldia magna*, have previously been detected in fecal samples from very low birth weight neonates (Brooks et al., 2014). An increased count of *F. magna* in stool samples of NEC infants was observed in the current study and a previous metagenome- and metaproteomics-based study (Brown et al., 2018). In addition, *F. magna* is detected in the amniotic fluid of women with preterm prelabor rupture of membranes (DiGiulio et al., 2010). Future studies are needed to determine the association between *F. magna* and NEC as well as the role of the inflammatory response in preterm delivery.

Limitations of this study include being unable to evaluate the impact of antibiotic exposure. All infants invariably received antibiotics while being admitted to the NICU, and 95% of them received broad-spectrum antibiotics, including meropenem and colistin. First-week samples were not available for six infants. An infant with NEC underwent surgical resection in the second week of life, and subsequent samples were not collected.

The current observational study using multiple statistical methods demonstrated that gestational age and probiotic supplementation influences the gut microbiota of preterm infants. Moderate preterm infants (>29 GA weeks) were associated with increased *Bifidobacterium breve* and reduced *Acinetobacter* compared with extremely preterm infants (<28 weeks). Probiotic supplementation increased *Bifidobacterium* and *B. thetaiotaomicron* and reduced the abundance of *Acinetobacter* as well as *Klebsiella* in infants admitted to the NICU. Finally, using limited samples, we showed a reduction in beneficial microbes in the gut of preterm infants with NEC. In conclusion, both gestational age and probiotic supplementation alter the gut microbiota of preterm infants admitted to the NICU.

## Data availability statement

The datasets presented in this study can be found in online repositories. The names of the repository/repositories and accession number(s) can be found below: NCBI–PRJNA1048725.

## Ethics statement

The studies involving humans were approved by the Institutional Ethics Committee, PGIMER. The studies were conducted in accordance with the local legislation and institutional requirements. Written informed consent for participation in this study was provided by the participants’ legal guardians/next of kin.

## Author contributions

PD: Data curation, Funding acquisition, Methodology, Investigation, Writing – original draft. JoK: Conceptualization, Data curation, Investigation, Writing – review and editing. SD: Conceptualization, Supervision, Writing – review and editing. SA: Conceptualization, Project administration, Supervision, Writing – review and editing. JaK: Conceptualization, Formal Analysis, Investigation, Methodology, Supervision, Validation, Writing – original draft, Writing – review and editing.
