# Supplementary material for: Gut microbiota of preterm infants in the neonatal intensive care unit: a study from a tertiary care center in northern India
Source: Front Microbiol. 2024 Feb 8;15:1329926. doi: 10.3389/fmicb.2024.1329926 (PMC10881769; doi:10.3389/fmicb.2024.1329926)
Supplement: Supplementary file 1 [file Data_Sheet_1.docx]

**Supplementary Material**

**Supplementary Table 1. PERMANOVA multivariate analysis was performed based on Jaccard dissimilarity distance.**

|  | **Df** | **SumsOfSqs** | **MeanSqs** | **F.Model** | **R2** | **Pr(>F)** |
| --- | --- | --- | --- | --- | --- | --- |
| **week** | 3 | 0.63158826 | 0.21052942 | 0.73976645 | 0.04018921 | 0.84 |
| **probiotics** | 1 | 0.97097026 | 0.97097026 | 3.41183302 | 0.06178476 | **0.01** |
| **preterm** | 1 | 0.96130094 | 0.96130094 | 3.37785658 | 0.06116948 | **0.01** |
| **NEC** | 1 | 0.56743401 | 0.56743401 | 1.99387169 | 0.03610695 | **0.04** |
| **week:probiotics** | 3 | 0.77380836 | 0.25793612 | 0.90634596 | 0.04923896 | 0.65 |
| **week:preterm** | 3 | 0.90961113 | 0.30320371 | 1.06540897 | 0.05788036 | 0.31 |
| **probiotics:preterm** | 1 | 0.57561515 | 0.57561515 | 2.02261888 | 0.03662753 | 0.06 |
| **week:NEC** | 2 | 0.66578692 | 0.33289346 | 1.16973397 | 0.04236534 | 0.28 |
| **probiotics:NEC** | 1 | 0.67851355 | 0.67851355 | 2.38418727 | 0.04317516 | **0.03** |
| **preterm:NEC** | 1 | 0.51037149 | 0.51037149 | 1.79336316 | 0.03247595 | **0.03** |
| **week:probiotics:preterm** | 3 | 0.97041657 | 0.32347219 | 1.13662915 | 0.06174953 | 0.38 |
| **week:probiotics:NEC** | 2 | 0.3852251 | 0.19261255 | 0.67680945 | 0.02451264 | 0.89 |
| Residuals | 25 | 7.11472581 | 0.28458903 | NA | 0.45272411 | NA |
| Total | 47 | 15.7153675 | NA | NA | 1 | NA |

**Supplementary Table 2. PERMANOVA multivariate analysis performed based on weighted UniFrac distance.**

|  | **Df** | **SumsOfSqs** | **F.Model** | **R2** | **Pr(>F)** |
| --- | --- | --- | --- | --- | --- |
| week | 3 | 0.2081 | 0.02946 | 0.4451 | 0.97 |
| probiotics | 1 | 0.6218 | 0.088 | 4.4388 | **0.004** |
| preterm | 1 | 0.6353 | 0.08991 | 4.5446 | **0.004** |
| NEC | 1 | 0.2308 | 0.03266 | 1.553 | 0.163 |

**Supplementary Figure 1. Graphical representation of time points for sample collection from preterm infants.**

**
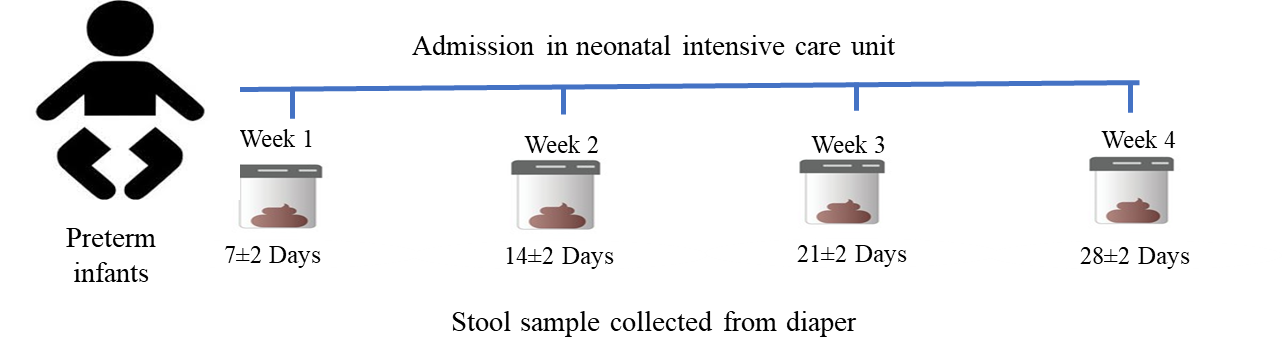
**

**Supplementary Figure 2. Alpha diversity metrics observed ASVs (A) and Shannon diversity (B) in extremely and moderate preterm infants. Observed ASVs (C) and Shannon diversity (D) in probiotic-supplemented and non-probiotic samples of preterm infants.**

A.


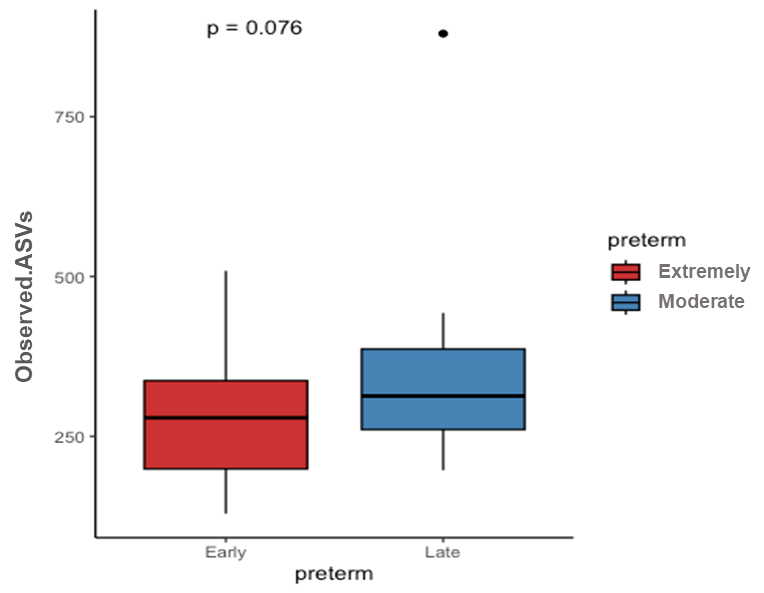


B.


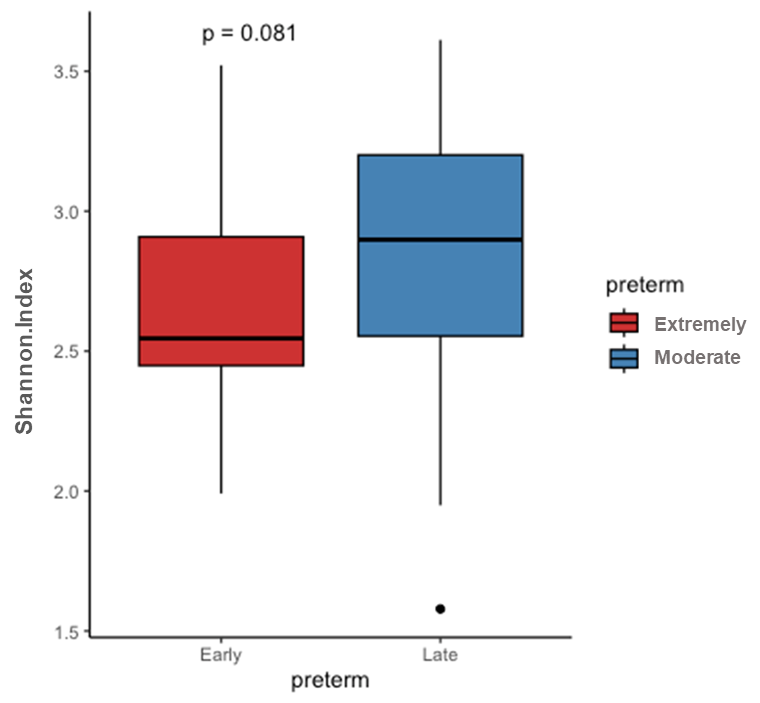


C.

**Observed.ASVs**


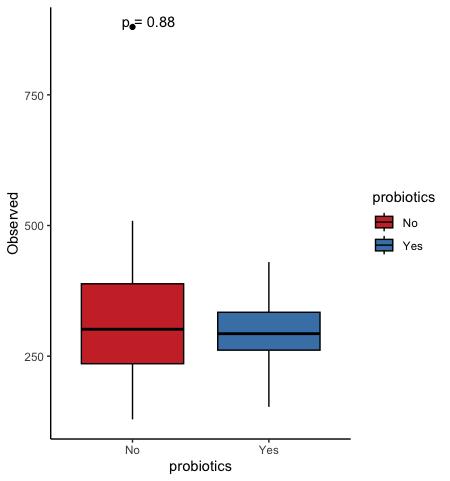


D.

**
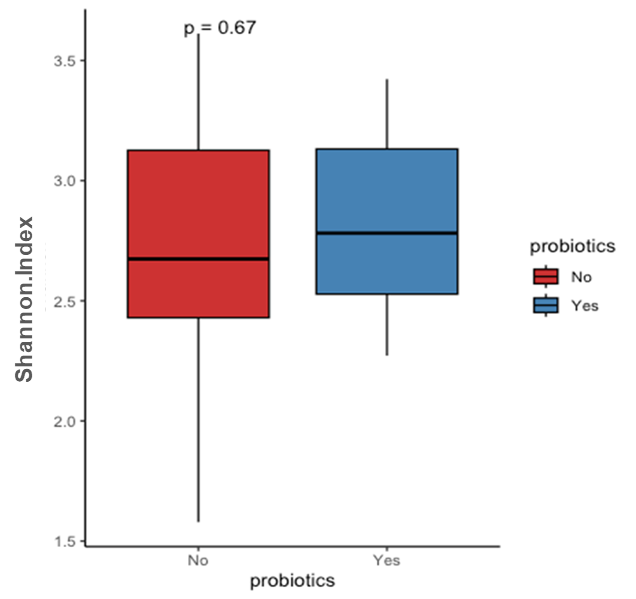
**

**Supplementary figure 3**. **Alpha diversity metrics observed ASVs (A) and Shannon diversity (B) in infants with and without NEC.**

A.

**Observed.ASVs**


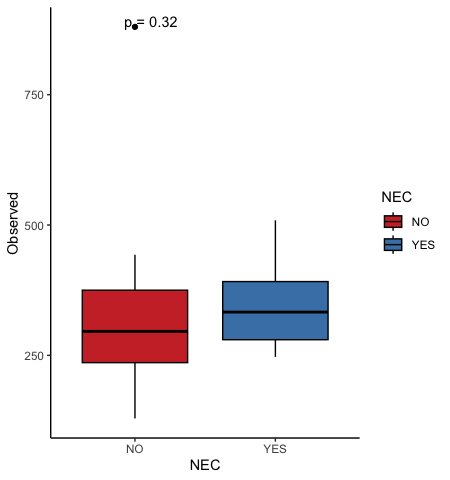


B.

**
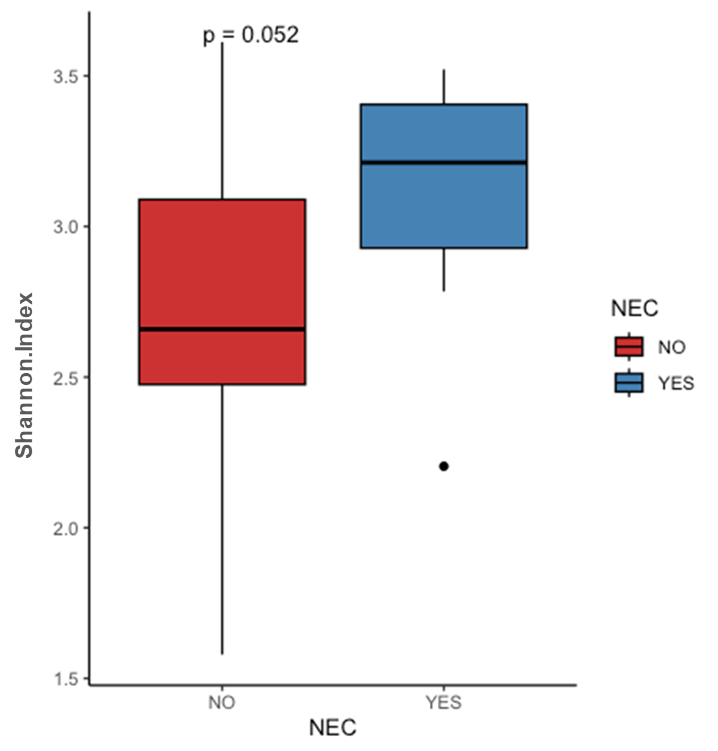
**

**Supplementary figure 4**. **LEfSe analysis for differential microbial abundance between samples after probiotic supplementation in infants and before or after no probiotic supplementation in infant samples.** Bacterial abundance shown only to significant corresponding Taxa level


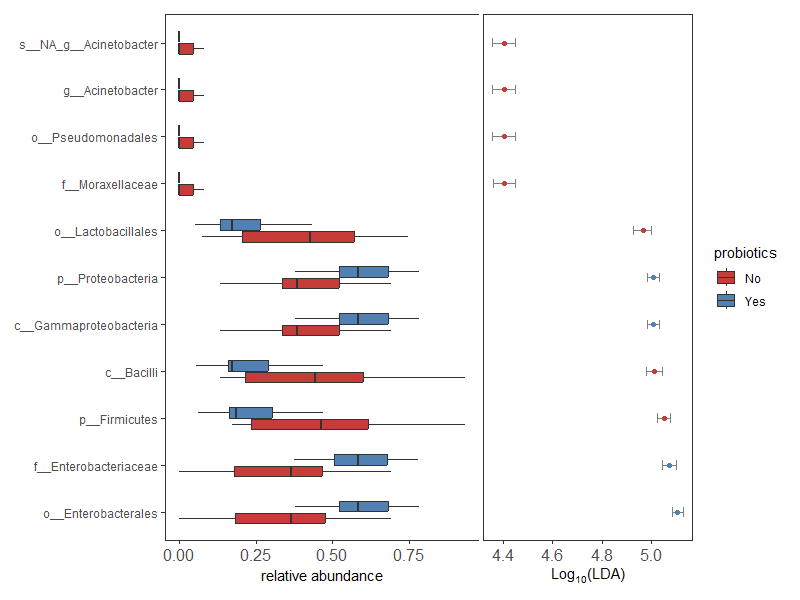


**Supplementary table 3. DESeq2 analysis for the differential bacterial abundance between** **extremely preterm infants (<29 weeks) and moderate preterm infants (29-32 weeks)**

|  | **baseMean** | **log2FoldChange** | **lfcSE** | **stat** | **pvalue** | **padj** | **Kingdom** | **Phylum** | **Class** | **Order** | **Family** | **Genus** | **Species** |
| --- | --- | --- | --- | --- | --- | --- | --- | --- | --- | --- | --- | --- | --- |
| ASV_11 | 1386.433 | 29.79412 | 1.741389 | 17.1094 | 1.26E-65 | 4.03E-63 | Bacteria | Actinobacteriota | Actinobacteria | Bifidobacteriales | Bifidobacteriaceae | Bifidobacterium | breve |
| ASV_14 | 1251.42 | 29.64946 | 1.741656 | 17.02372 | 5.48E-65 | 8.74E-63 | Bacteria | Actinobacteriota | Actinobacteria | Bifidobacteriales | Bifidobacteriaceae | Bifidobacterium | breve |
| ASV_18 | 489.0217 | 28.31038 | 2.942064 | 9.622626 | 6.42E-22 | 4.09E-20 | Bacteria | Actinobacteriota | Actinobacteria | Bifidobacteriales | Bifidobacteriaceae | Bifidobacterium | longum |
| ASV_29 | 508.112 | 28.37005 | 2.642362 | 10.73663 | 6.85E-27 | 6.01E-25 | Bacteria | Proteobacteria | Gammaproteobacteria | Enterobacterales | Enterobacteriaceae | Escherichia-Shigella | NA |
| ASV_30 | 474.6054 | 28.28861 | 2.636938 | 10.72782 | 7.53E-27 | 6.01E-25 | Bacteria | Proteobacteria | Gammaproteobacteria | Enterobacterales | Enterobacteriaceae | Escherichia-Shigella | NA |
| ASV_47 | 8.958448 | 22.73984 | 2.942876 | 7.727083 | 1.10E-14 | 4.39E-13 | Bacteria | Firmicutes | Clostridia | Clostridiales | Clostridiaceae | Clostridium sensu stricto 1 | butyricum |
| ASV_49 | 8.162167 | 20.97735 | 2.942956 | 7.127984 | 1.02E-12 | 1.91E-11 | Bacteria | Firmicutes | Clostridia | Clostridiales | Clostridiaceae | Clostridium sensu stricto 1 | butyricum |
| ASV_65 | 6.523135 | 22.30681 | 2.943183 | 7.579145 | 3.48E-14 | 1.01E-12 | Bacteria | Bacteroidota | Bacteroidia | Bacteroidales | Bacteroidaceae | Bacteroides | thetaiotaomicron |
| ASV_69 | 6.661086 | 22.33675 | 2.94316 | 7.589377 | 3.21E-14 | 1.01E-12 | Bacteria | Bacteroidota | Bacteroidia | Bacteroidales | Bacteroidaceae | Bacteroides | thetaiotaomicron |
| ASV_71 | 52.15459 | -10.6428 | 2.934901 | -3.62628 | 0.000288 | 0.005096 | Bacteria | Firmicutes | Clostridia | Peptostreptococcales-Tissierellales | Family XI | Finegoldia | magna |
| ASV_84 | 5.685536 | 22.10617 | 2.94335 | 7.510546 | 5.89E-14 | 1.25E-12 | Bacteria | Proteobacteria | Gammaproteobacteria | Enterobacterales | Yersiniaceae | Serratia | marcescens |
| ASV_87 | 6.231535 | 22.24243 | 2.943237 | 7.557131 | 4.12E-14 | 1.10E-12 | Bacteria | Proteobacteria | Gammaproteobacteria | Enterobacterales | Yersiniaceae | Serratia | marcescens |
| ASV_92 | 4.177959 | 21.68663 | 2.943818 | 7.366837 | 1.75E-13 | 3.48E-12 | Bacteria | Bacteroidota | Bacteroidia | Bacteroidales | Bacteroidaceae | Bacteroides | NA |
| ASV_97 | 8.984101 | 22.15892 | 2.942873 | 7.52969 | 5.09E-14 | 1.16E-12 | Bacteria | Proteobacteria | Gammaproteobacteria | Enterobacterales | Yersiniaceae | Serratia | NA |
| ASV_99 | 8.946699 | 22.74776 | 2.942877 | 7.72977 | 1.08E-14 | 4.39E-13 | Bacteria | Proteobacteria | Gammaproteobacteria | Enterobacterales | Yersiniaceae | Serratia | NA |
| ASV_103 | 6.622771 | 22.33021 | 2.943166 | 7.587137 | 3.27E-14 | 1.01E-12 | Bacteria | Proteobacteria | Gammaproteobacteria | Enterobacterales | Yersiniaceae | Serratia | NA |
| ASV_106 | 5.971425 | 22.18365 | 2.943288 | 7.53703 | 4.81E-14 | 1.16E-12 | Bacteria | Proteobacteria | Gammaproteobacteria | Enterobacterales | Yersiniaceae | Serratia | NA |
| ASV_155 | 7.018167 | -24.3227 | 2.937113 | -8.28114 | 1.22E-16 | 6.49E-15 | Bacteria | Proteobacteria | Gammaproteobacteria | Pseudomonadales | Moraxellaceae | Acinetobacter | NA |

**Supplementary table 4. DESeq2 analysis for differential bacterial abundance between samples after probiotic administration in infants and** before/no probiotic administration in infant samples.

|  | **baseMean** | **log2FoldChange** | **lfcSE** | **stat** | **pvalue** | **padj** | **Kingdom** | **Phylum** | **Class** | **Order** | **Family** | **Genus** | **Species** |
| --- | --- | --- | --- | --- | --- | --- | --- | --- | --- | --- | --- | --- | --- |
| ASV_9 | 1981.126 | -8.91472 | 1.630001 | -5.46915 | 4.52E-08 | 1.64E-05 | Bacteria | Proteobacteria | Gammaproteobacteria | Pseudomonadales | Moraxellaceae | Acinetobacter | NA |
| ASV_10 | 1958.177 | -8.70946 | 1.601919 | -5.43689 | 5.42E-08 | 1.84E-05 | Bacteria | Proteobacteria | Gammaproteobacteria | Pseudomonadales | Moraxellaceae | Acinetobacter | NA |
| ASV_17 | 47.53023 | 26.72493 | 3.081735 | 8.672043 | 4.24E-18 | 5.76E-15 | Bacteria | Actinobacteriota | Actinobacteria | Bifidobacteriales | Bifidobacteriaceae | Bifidobacterium | longum |
| ASV_18 | 39.36573 | 26.3663 | 3.08177 | 8.555572 | 1.17E-17 | 9.59E-15 | Bacteria | Actinobacteriota | Actinobacteria | Bifidobacteriales | Bifidobacteriaceae | Bifidobacterium | longum |
| ASV_30 | 207.9505 | 28.44367 | 3.081604 | 9.230152 | 2.70E-20 | 4.89E-17 | Bacteria | Proteobacteria | Gammaproteobacteria | Enterobacterales | Enterobacteriaceae | Escherichia-Shigella | NA |
| ASV_50 | 416.6557 | -10.5752 | 2.057038 | -5.14098 | 2.73E-07 | 8.25E-05 | Bacteria | Proteobacteria | Gammaproteobacteria | Enterobacterales | Enterobacteriaceae | Klebsiella | NA |
| ASV_51 | 406.8198 | -12.73 | 2.021553 | -6.29712 | 3.03E-10 | 1.18E-07 | Bacteria | Proteobacteria | Gammaproteobacteria | Enterobacterales | Enterobacteriaceae | Klebsiella | NA |
| ASV_60 | 324.3684 | -10.703 | 2.02183 | -5.29373 | 1.20E-07 | 3.83E-05 | Bacteria | Proteobacteria | Gammaproteobacteria | Enterobacterales | Enterobacteriaceae | Klebsiella | NA |
| ASV_64 | 315.2516 | -27.8516 | 2.052488 | -13.5697 | 6.06E-42 | 3.29E-38 | Bacteria | Proteobacteria | Gammaproteobacteria | Enterobacterales | Enterobacteriaceae | Klebsiella | NA |
| ASV_65 | 6.523135 | 23.9432 | 3.082797 | 7.766714 | 8.05E-15 | 4.37E-12 | Bacteria | Bacteroidota | Bacteroidia | Bacteroidales | Bacteroidaceae | Bacteroides | thetaiotaomicron |
| ASV_69 | 6.661086 | 23.97358 | 3.082772 | 7.776632 | 7.45E-15 | 4.37E-12 | Bacteria | Bacteroidota | Bacteroidia | Bacteroidales | Bacteroidaceae | Bacteroides | thetaiotaomicron |
| ASV_91 | 3.192592 | 22.94876 | 3.084079 | 7.441044 | 9.99E-14 | 4.52E-11 | Bacteria | Bacteroidota | Bacteroidia | Bacteroidales | Bacteroidaceae | Bacteroides | thetaiotaomicron |
| ASV_92 | 4.177959 | 23.32424 | 3.083487 | 7.564241 | 3.90E-14 | 1.93E-11 | Bacteria | Bacteroidota | Bacteroidia | Bacteroidales | Bacteroidaceae | Bacteroides | NA |
| ASV_96 | 39.6442 | 26.34769 | 3.081768 | 8.549538 | 1.24E-17 | 9.59E-15 | Bacteria | Bacteroidota | Bacteroidia | Bacteroidales | Bacteroidaceae | Bacteroides | thetaiotaomicron |
| ASV_98 | 3.35025 | 21.52823 | 3.083962 | 6.980707 | 2.94E-12 | 1.23E-09 | Bacteria | Bacteroidota | Bacteroidia | Bacteroidales | Bacteroidaceae | Bacteroides | NA |
| ASV_100 | 39.82344 | 26.47445 | 3.081767 | 8.590672 | 8.65E-18 | 9.39E-15 | Bacteria | Bacteroidota | Bacteroidia | Bacteroidales | Bacteroidaceae | Bacteroides | NA |
| ASV_107 | 35.93005 | 26.21979 | 3.081789 | 8.507978 | 1.77E-17 | 1.20E-14 | Bacteria | Bacteroidota | Bacteroidia | Bacteroidales | Bacteroidaceae | Bacteroides | NA |
| ASV_163 | 43.10058 | -25.1254 | 2.188357 | -11.4814 | 1.64E-30 | 4.44E-27 | Bacteria | Proteobacteria | Gammaproteobacteria | Enterobacterales | Enterobacteriaceae | NA | NA |

**Supplementary table 5. LEfSe analysis of the gut microbiota abundance of the microbiome between preterm infants with and without NEC**

| f | **LDAupper** | **LDAmean** | **LDAlower** | **NEC** | **pvalue** |
| --- | --- | --- | --- | --- | --- |
| g__Finegoldia | 3.741527 | 3.662923 | 3.566878 | YES | 0.000252 |
| s__magna | 3.708903 | 3.631561 | 3.537397 | YES | 0.000252 |

**Supplementary table 6. DESeq2 analysis for differential bacterial abundance between preterm infants with and without NEC**

|  | **baseMean** | **log2FoldChange** | **lfcSE** | **stat** | **pvalue** | **padj** | **Kingdom** | **Phylum** | **Class** | **Order** | **Family** | **Genus** | **Species** |
| --- | --- | --- | --- | --- | --- | --- | --- | --- | --- | --- | --- | --- | --- |
| ASV_17 | 106.5874 | -25.5066 | 4.159778 | -6.13172 | 8.69E-10 | 1.66E-07 | Bacteria | Actinobacteriota | Actinobacteria | Bifidobacteriales | Bifidobacteriaceae | Bifidobacterium | longum |
| ASV_18 | 96.11003 | -25.3333 | 4.159787 | -6.09005 | 1.13E-09 | 1.79E-07 | Bacteria | Actinobacteriota | Actinobacteria | Bifidobacteriales | Bifidobacteriaceae | Bifidobacterium | longum |
| ASV_65 | 6.523135 | -21.6653 | 4.16108 | -5.20666 | 1.92E-07 | 1.53E-05 | Bacteria | Bacteroidota | Bacteroidia | Bacteroidales | Bacteroidaceae | Bacteroides | thetaiotaomicron |
| ASV_69 | 6.661086 | -21.7329 | 4.161052 | -5.22293 | 1.76E-07 | 1.53E-05 | Bacteria | Bacteroidota | Bacteroidia | Bacteroidales | Bacteroidaceae | Bacteroides | thetaiotaomicron |
| ASV_70 | 11.80407 | -22.4385 | 3.38477 | -6.62927 | 3.37E-11 | 8.05E-09 | Bacteria | Firmicutes | Bacilli | Staphylococcales | Staphylococcaceae | Staphylococcus | epidermidis |
| ASV_95 | 16.48421 | -22.9596 | 4.105047 | -5.59302 | 2.23E-08 | 3.04E-06 | Bacteria | Firmicutes | Bacilli | Staphylococcales | Staphylococcaceae | Staphylococcus | NA |
| ASV_97 | 8.984101 | -21.4019 | 4.160703 | -5.14383 | 2.69E-07 | 1.98E-05 | Bacteria | Proteobacteria | Gammaproteobacteria | Enterobacterales | Yersiniaceae | Serratia | NA |
| ASV_99 | 8.946699 | -22.1226 | 4.160707 | -5.31703 | 1.05E-07 | 1.12E-05 | Bacteria | Proteobacteria | Gammaproteobacteria | Enterobacterales | Yersiniaceae | Serratia | NA |
| ASV_102 | 98.46315 | -25.0386 | 2.713242 | -9.2283 | 2.75E-20 | 1.31E-17 | Bacteria | Proteobacteria | Gammaproteobacteria | Enterobacterales | Enterobacteriaceae | NA | NA |
| ASV_103 | 6.622771 | -21.7269 | 4.16106 | -5.22148 | 1.78E-07 | 1.53E-05 | Bacteria | Proteobacteria | Gammaproteobacteria | Enterobacterales | Yersiniaceae | Serratia | NA |
| ASV_108 | 102.4454 | -25.4366 | 2.440826 | -10.4213 | 1.98E-25 | 1.89E-22 | Bacteria | Proteobacteria | Gammaproteobacteria | Enterobacterales | Enterobacteriaceae | NA | NA |
| ASV_163 | 43.10058 | -24.2292 | 3.144549 | -7.70516 | 1.31E-14 | 4.16E-12 | Bacteria | Proteobacteria | Gammaproteobacteria | Enterobacterales | Enterobacteriaceae | NA | NA |
| ASV_169 | 9.966574 | -22.2773 | 4.160605 | -5.35435 | 8.59E-08 | 1.02E-05 | Bacteria | Firmicutes | Bacilli | Staphylococcales | Staphylococcaceae | Staphylococcus | epidermidis |

**Supplementary table 7. MaAsLin2 linear model multivariate analysis adjusted for postnatal age for altered the gut microbiota of preterm infants**

| **feature** | **metadata** | **value** | **coefficient** | **stderr** | **N** | **p value** | **q value** |
| --- | --- | --- | --- | --- | --- | --- | --- |
| \| *Bifidobacterium* \| *Longum* \| \| --- \| --- \| | preterm | moderate | 5.80370181 | 1.27427969 | 48 | 3.99E-05 | 0.01393698 |
| *Bacteroides thetaiotaomicron* | preterm | moderate | 6.21339178 | 1.34281682 | 48 | 3.15E-05 | 0.01393698 |
| \| *Bifidobacterium* \| *Breve* \| \| --- \| --- \| | probiotics | Yes | 2.19668482 | 0.48540432 | 48 | 4.38E-05 | 0.01393698 |
| *Bacteroides thetaiotaomicron* | probiotics | Yes | 2.15537241 | 0.49399682 | 48 | 7.40E-05 | 0.01487725 |
| *Acinetobacter* | probiotics | Yes | -2.480191 | 0.57057424 | 48 | 7.80E-05 | 0.01487725 |
